# Bayesian decision-making under stress-preserved weighting of prior and likelihood information

**DOI:** 10.1038/s41598-020-76493-5

**Published:** 2020-12-08

**Authors:** Sabrina Trapp, Iris Vilares

**Affiliations:** 1 Department of Psychology, University of Hamburg, Hamburg, Germany; 2 Department of Psychology, University of Minnesota, Minneapolis, MN USA; 3 Faculty of Psychology and Sports Science, Bielefeld University, Bielefeld, Germany

**Keywords:** Psychology, Human behaviour

## Abstract

A rich body of empirical work has addressed the question of how stress changes the way we memorize, learn, and make high-level decisions in complex scenarios. There is evidence that stress also changes the way we perceive the world, indicating influences on decision-making at lower levels. Surprisingly, as of yet, little research has been conducted in this domain. A few studies suggest that under stress, humans tend to eschew existing knowledge, and instead focus on novel input or information from bottom-up. Decision-making in the perceptual domain has been modeled with Bayesian frameworks. Here, existing knowledge about structures and statistics of our environment is referred to as *prior*, whereas sensory data are termed *likelihood*. In this study, we directly assessed whether stress, as induced by the socially evaluated cold pressure task (SECPT), would modulate low-level decisions, specifically the weight given to sensory information, and how people reacted to changes in prior and sensory uncertainty. We found that while the stress-inducing procedure successfully elicited subjective stress ratings as well as stress relevant physiological paramters, it did not change participants’ average reliance on sensory information. Furthermore, it did not affect participants’ sensitivity to changes in prior and sensory uncertainty, with both groups able to detect it and modulate their behavior accordingly, in a way predicted by Bayesian statistics. Our results suggest that, contrary to our predictions, stress may not directly affect lower-level sensory-motor decisions. We discuss the findings in context of time scales of the stress reaction, linked to different neural and functional consequences.

## Introduction

Stress is a ubiquitous phenomenon in our world, and its effects on health and cognition, the underlying neural networks, and physiological mechanisms are subject to intense empirical investigations. Enhanced memory for stressful events as well as impaired retrieval from long-term memory has been in the focus of scientific endeavors^1^. Additionally, a plethora of studies examined modifications of working memory functioning during stress and current evidence points to an impairment in memorizing over the short-term^2,3^. Possibly, stress impairs functioning of this faculty by altered processing of Dopamine 1 (DA1) receptors induced by glucocorticoids^4^. The DA1 receptors seem to be vital for prefrontal cortex (PFC) functioning, presumably for task-related, reverberatory activity during the maintenance of information in animals and humans^5,6^. However, the question of whether and how low-level decision-making is affected has been much less explored. In the locus coeruleus, a shift from a phasic towards a tonic mode of activity has been reported under stress, thus in principle able to promote enhanced saliency detection, possibly via an increase in sensory gating capacity^7^. When exposed to a rapid stream of sensory data, participants usually miss a second target that is presented maximally 500 ms after the first one, referred to as the *attentional blink*^8^. This effect has been explained by attention being allocated to the processing of the first target, thus leaving no capacity to process the second target. The attentional blink is reduced under conditions of stress^9^. This may be linked to a generally enhanced (in this context preserved) ability to process incoming sensory information, i.e., data from bottom-up, possibly at costs for top-down or prior information residing within the system. Another important finding is that in situations of acute stress, the functioning of the PFC is affected, possibly because the system switches to a bottom-up control by the sensory cortices^10^. Stress is also associated with impaired memory retrieval, as explained by the operation of glucocorticoids in dedicated brain areas^11^. An alternative, functional account of these effects may be that previously stored, prior information is no longer useful, and consequently, its access (adaptively) impeded. This may equal a cognitive strategy that actively eschews stored generative models about the environment, and instead gives more weight to sensory data, i.e., incoming information. There is also evidence that acute stress increases exploration and risky decision-making^12,13^. Possibly, this corresponds to a strategy that actively seeks new, possibly better models of the environment.

An enhanced focus on saliency would support the stressed organism in quickly identifying vital and important information in the environment. However, evidence for such a re-allocation of resources in the top-down versus bottom-up dynamics remains scarce. To test this idea, we translate the top-down/bottom-up dichotomy to a Bayesian framework. In essence, the Bayesian framework allows formalizing how the brain combines existing information relevant for the decision, the *prior* (i.e., expectations delivered from ‘top’) with actual sensory input, the *likelihood* (i.e., information from the ‘bottom’) to achieve fast and accurate inferences^14,15^. It adopts a normative approach, i.e., specifies what would be the optimal inference given the data, so to make the best possible decision. Although in practice people are not perfectly optimal, comparing what people “should” do with what people actually do allows us to better understand the biases and information that people have available^16^.

To directly assess whether stress induces a putative shift from prior knowledge to incoming sensory data (i.e., the likelihood), we applied a visual decision-making task (used in previous studies^17^) and manipulated prior and likelihood uncertainty, respectively. Participants were randomly assigned to a stressed or a not-stressed control group, and data were fitted with Bayesian models to discover possible changes in the way people use information. One would expect that, if acute stress increases reliance on sensory information, this would show as an increased prior variance in the stressed group (given that a higher prior variance in the Bayesian model would lead to higher reliance on sensory information). The data were in line with previous findings, indicating optimal sensory-motor decision-making consistent with Bayesian statistics^18^. However, and contrary to our expectations, we did not find that stress lead to an increasing reliance on incoming sensory information.

## Results

### A priori group differences and manipulation check

**Figure 1:**
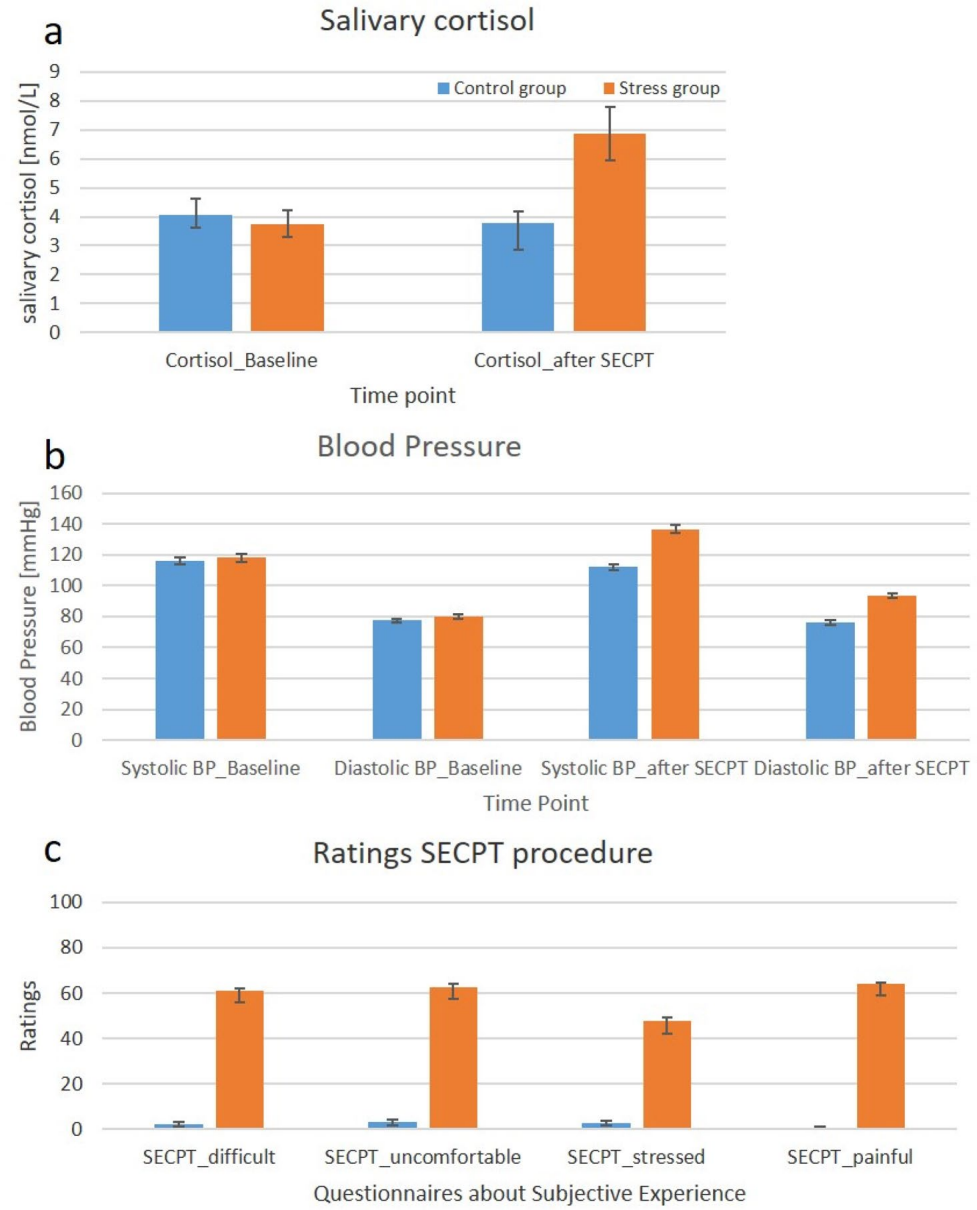
Effect of the stress manipulation (SECPT). Represented are the average ± standard error of the (**a**) salivary cortisol levels, and (**b**) systolic and diastolic blood pressure, at baseline and after the stress-induction procedure (the socially evaluated cold pressure task, SECPT). Participants (n = 60) were randomly allocated to two groups: The control group and the stressed group. Participants in the stressed group received the SECPT procedure. Namely, they had to immerse their hand into ice water (0–2 °C) and keep it there for 3 min. Additionally, they had to look into a camera where they were being observed by a non-supportive, unfriendly experimenter wearing a white coat. The control group, on the other hand, immersed their hand in warm water (35–37 °C), did not have to look into a camera, and the experimenter was casually dressed and friendly (see “Methods”). (**c**) Self-reported ratings of the SECPT procedure.

To ensure that the groups did not a priori differ in subjective mood, salivary cortisol, and systolic or diastolic blood pressure, we compared these values across groups before the beginning of the experiment. The data showed no significant differences between the two groups (*d* = 0.06 Cohen’s d effect size, *W*(29,29) = 827, *p* = 0.67 for positive subjective mood; *d* = 0.14, *W*(31,29) = 859, *p* = 0.2 for negative mood; *d* = − 0.116, *W*(31,29) = 950, *p* = 0.95 for baseline cortisol differences, *d* = 0.17, *t*(58) = 0.66, *p* = 0.51 for baseline systolic blood pressure , *d* = 0.33, *t*(58) = 1.27, *p* = 0.21 for baseline diastolic blood pressure; see Fig. 1). Moreover, there were no group differences in state or trait anxiety (*d* = − 0.04, *t*(58) = 0.15, *p* = 0.88), depressive mood (*d* = − 0.18, *W*(31,29) = 863, *p* = 0.736), perceived chronic stress (*d* = 0.28, *t*(56) = 1.06, p = 0.29), or social anxiety (*d* = − 0.02, *W*(31,29) = 910, *p* = 0.60). As a manipulation check, i.e., whether the stress induction was successful, increases in salivary cortisol after the stress induction were compared between the stressed group as opposed to the control group. The data showed a significantly higher cortisol increase in the stressed group (mean changes in cortisol: *M* = 3.13 nmol/L, *SD* = 5.35, *n* = 29) compared to the control group (*M* = − 0.28 nmol/L, *SD* = 1.98, *n* = 31; *d* = 0.86, *W*(31,29) = 748, *p* = 0.003; see Fig. 1a), leading both groups to differ significantly in their salivary cortisol levels after the stress induction (*d* = 0.82, *W*(31,29) = 771, *p* = 0.01). The stressed group also had larger average changes in the systolic (*M* = 19.2, *SD* = 12 vs. *M* = − 3.68, *SD* = 6.8 mmHg, *d* = 2.39, *t*(57) = 9.15, *p* < 10^–12^) and diastolic blood pressure (*M* = 13.7, *SD* = 8.4 vs. *M* = − 1.35, *SD* = 3.7 mmHg, *d* = 2.37, *t*(57) = 9.08, p < 10^–11^; see Fig. 1b). This resulted in a higher average systolic and diastolic blood pressure in the stressed group after the stress manipulation compared to the control group (137/94 mmHg vs. 112/76 mmHg, *d* = 2.06, *t*(57) = 7.9, *p* < 10^–10^ for systolic; *d* = 2.23, *W*(31,29) = 547, *p* < 10^–10^ for diastolic blood pressure; see Fig. 1b). Subjectively, when participants were asked to rate what they felt about the stress procedure they had just experienced, participants in the stressed group rated it as more difficult (*d* = 2.96, *W*(31,29) = 555, *p* < 10^–10^), uncomfortable (*d* = 2.79, *W*(31,29) = 538, *p* < 10^–11^), stressful (*d* = 2.06, *W*(31,29) = 593, *p* < 10^–8^) and painful (*d* = 3.26, *W*(31,29) = 500.5, *p* < 10^–15^; see Fig. 1c). These self-report feelings of stress/discomfort associated with the procedure, together with the significant selective increases in biological measures of stress only for the stressed group, indicate that our stress induction procedure was effective.

### Stress and Bayesian decision-making

**Figure 2:**
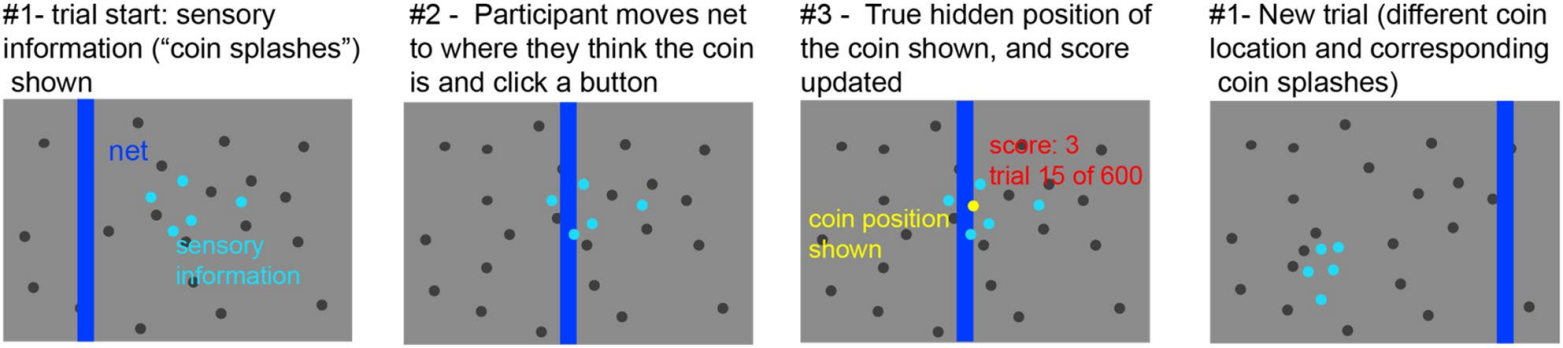
Experimental procedure. In this task, participants have to guess the hidden position of a target based on noisy information. They are told that they have to find a coin (yellow dot, which is hidden) and place the net (blue vertical bar) where they think the coin is. Furthermore, they are told that the person throwing the coin is aiming at the center. At the beginning of each trial, they are shown a cloud of light-blue dots and told that these are the splashes that the coin makes when it falls. Participants can move the blue net to where they think the coin is, and then press a button signaling their decision. Once they make the decision, they are shown the actual position of the coin in that trial, and get a point added to their score if the net covers at least half of the coin. The position of the coin is drawn from a Gaussian distribution (the “prior”) where the mean is the center of the screen and the variance is either large or small (P, σ^2^_P_ = 0.085^2^, and p, σ^2^_p_ = 0.025^2^, respectively, in unit-less screen coordinates). The sensory information, or likelihood, consists of 5 light blue dots which are taken from another Gaussian distribution where the mean is the position of the coin at that trial, and the variance is either large (L, σ^2^_l_ = 0.15^2^) or small (σ^2^_l_ = 0.06^2^). The experiment is divided in blocks of 150 trials each, and within a block the prior remains constant. Each prior type is shown twice, for a total of 4 blocks (2 types of prior variance * 2 block repetitions) and 600 trials (150*4). The sensory information varies from trial to trial, pseudo-randomly (so that there are 75 trials of each type of likelihood per prior block). This task is identical to the one used in^17^. For more details, see “Methods”.

**Figure 3:**
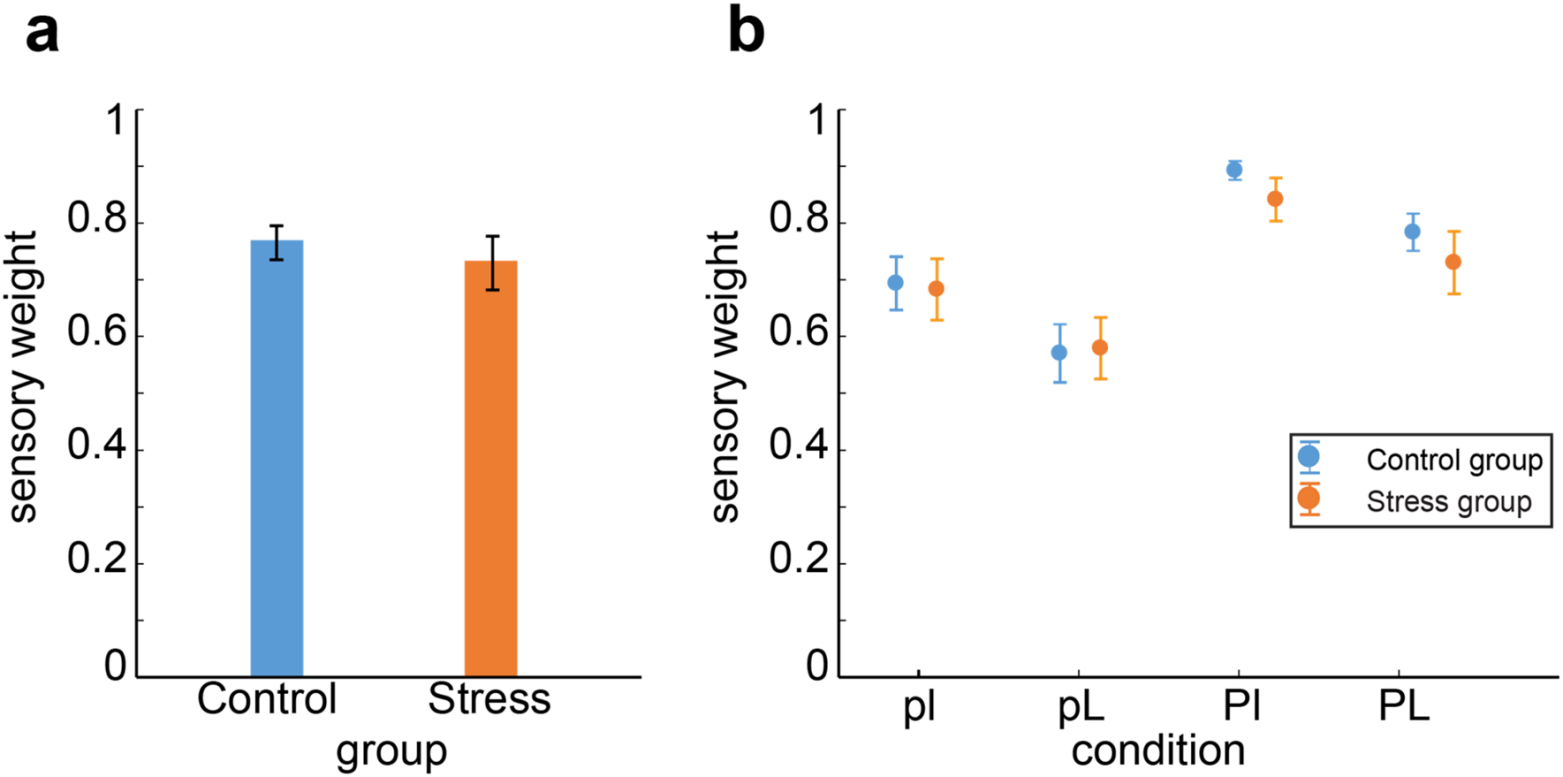
Behavioral results. (**a**) Average general sensory weight for participants in the stressed group (in orange) and the control group (in blue). The sensory weight quantifies the degree to which subjects rely on the current visual sensory information (likelihood) vs. the prior (see “Methods” for details). (**b**) Average sensory weights, separated by condition. Error bars in (**a**) and (**b**) represent the standard error of the mean. Conditions: pl: small prior and sensory/likelihood uncertainty; pL: small prior uncertainty, large sensory uncertainty; Pl: large prior uncertainty, small sensory uncertainty; PL: large prior and sensory uncertainty.

To analyze if stress increased the relative weight given to sensory information, we looked at how participant’s estimated positions of the target coin location at each trial was driven by the sensory information (likelihood) about the target coin in that trial, and if this changed between the stressed and not-stressed groups (see Figs. 2 and 3). For this, we did a linear regression per participant, trying to predict the participant’s estimated position of the target coin as a function of the centroid of the cloud of dots shown in each trial^18^. The slope of this regression, the *sensory weight*, represents the relative weight a participant gives to sensory information, and should be near 1 if participants only rely on current sensory information and closer to 0 if they do not rely on it (for example, if they rely mainly on prior information; see SI for details). If we assume that participants use only current sensory information or prior information, then the weight on prior information is just *1—sensory weight*^19^. Our hypothesis was that the stress induction would make participants in the stressed group increase their attention and reliance in current sensory input, and hence have larger sensory weights (closer to 1) compared to the control group. For both groups the slopes of the linear regression (sensory weights) were significantly different from zero (control group: mean weight *M* = 0.764, *SD* = 0.166, *Mdn* = 0.776, *T*(31) = 496, *p* < 10^–5^; stressed group: *M* = 0.729, *Mdn* = 0.789, T(29) = 432, *p* < 10^–5^, Wilcoxon signed-rank test; see Fig. 3), indicating that both groups used sensory information to estimate the target’s position. The sensory weights were also significantly different from one, suggesting that they did not exclusively rely on current sensory information (Wilcoxon signed-rank test, T(31 or 29) = 0, *p* < 10^–5^, as there were essentially no cases in which a participant’s general sensory weight was at or above 1). However, and contrary to our predictions, we did not find a difference in the average sensory weights between the control and the stressed groups (*d* = − 0.16 Cohen’s d effect size*, W*(31,29) = 948, *p* = 0.976, Wilcoxon rank-sum test; see Fig. 3a). If we do multilevel modelling directly testing the (fixed) effects of stress on the sensory weight, with random effects accounting for participant-level variation, we find a significant effect of sensory information (sensory weight different from 0, *p* = 0) but no significant effect of stress on the sensory weight (*p* = 0.52) in predicting participants’ estimated coin position (see SI for details). Thus, in our sample, we did not find evidence that stress affects the relative weight given to sensory information.

We were also interested in looking at the impact of stress on participants’ ability to react to changes in sensory and/or prior information. If stress increases attention to incoming sensory information, then one might expect participants in the stress condition to also be better able to detect and react to changes in sensory uncertainty. To analyze this, we calculated the sensory weights separated by the four experimental conditions (pl: small prior and sensory/likelihood uncertainty; pL: small prior uncertainty, large sensory uncertainty; Pl: large prior uncertainty, small sensory uncertainty; PL: large prior and sensory uncertainty; see “Methods”). We found that both prior uncertainty and likelihood uncertainty significantly affected participants’ sensory weights, with participants relying more on sensory information when the sensory information was more reliable or the prior information more uncertain (*F*(1,59) = 62.3, *p* < 10^–10^ for the main effect of prior uncertainty; *F*(1,59) = 103.1, *p* < 10^–13^ for main likelihood effect, repeated-measures ANOVA with prior, likelihood and group as fixed factors; Fig. 3b). This behavior is in accordance with what would be qualitatively predicted by a Bayesian-optimal decision-maker^16,19^. However, neither did we find a significant main effect of group (*F*(1,59) = 0.21, *p* = 0.647), nor any interaction effects (*F*(1,59) < 1.3, *p* > 0.25). This suggests that both groups were able to detect changes in prior and likelihood uncertainty and react to it in a way qualitatively predicted by Bayesian statistics.

We can also directly test for participants’ sensitivity to changes in prior and likelihood uncertainty, and how it may have been affected by the experimental manipulation. We found that both groups were sensitive to changes in prior and likelihood uncertainty *(*sensitivity to prior: T(31,control) = 485, *p* < 10^–5^ Wilcoxon signed-rank, t(28, stressed) = 5.47, *p* < 10^–5^; sensitivity to likelihood: t(30,control) = 8.22, *p* < 10^–8^, t(28, stressed) = 6.31, *p* < 10^–6^, one-sample t-test). However, there was no significant difference between the stressed and control groups (*d* = − 0.291, *W*(31,29) = 1012, *p* = 0.329, Wilcoxon rank-sum, for differences in sensitivity to prior uncertainty; *d* = − 0.102 t(58) = 0.39, *p* = 0.695, two-sample t-test, for likelihood/sensory uncertainty). Altogether, we did not find evidence that stress selectively affects participants’ reaction to uncertainty in sensory and/or prior information.

Finally, there were no significant differences between the groups in the general sensory weights even using only the first 50 trials (*d* = − 0.278, *W*(31,29) = 1037, *p* = 0.178, Wilcoxon rank-sum test), nor differences in performance (d = − 0.42, *W*(31,29) = 1048, *p* = 0.13), or in reaction times (*d* = − 0.35, *W*(31,29) = 1008, *p* = 0.36, Wilcoxon rank-sum test). See SI for additional analysis.

### Power analysis

One potential reason why we did not see an effect of stress could be lack of power. With our sample size (n = 31 for the control group and n = 29 for the stressed group) and a significance level of α = 0.05, we had a power of 0.48 to detect a medium effect size (d = 0.5) difference between two independent means, if we consider a two-tailed test, and a power of 0.61 for a one-tailed test^20^. Given our sample size, we should have been able to see an effect size of d = 0.74 with α = 0.05, and a power = 0.8 even in a two-tailed test (and be able to detect a large effect size, d = 0.8, with a power = 0.86^20^). This suggests that any potential effect of stress on the sensory weights, if there is one, is not a large effect.

## Model fitting

### General models

The sensory weight results suggest that participants behaved in a way qualitatively predicted by Bayesian statistics. To understand if these values are also close to the quantitative *Bayesian optimal* predictions, or if other models would better explain the data, we fit a series of models and looked at which model fit better, both overall and per group. Analyzing if or in which way participants differed from the optimum prescribed by Bayesian statistics, or if, instead, simple heuristics would better explain the data, can give us an idea of the types of information that participants have available to them. Furthermore, comparing these model fits across the stressed and not-stressed control groups allows us to analyze potential ways in which (acute) stress could affect decision-making.

Model fit was measured both by analyzing each model’s Bayesian Information Criterion (BIC), to choose the best model that also penalized unnecessary parameters, but also by checking which model made better cross-validated predictions. To obtain the cross-validated predictions, we fit each model to the *odd* trials of a participant’s data and tested it on the *even* trials. The resulting (root) mean squared cross-validated errors associated with each model were then used to compare model fits across groups.

Bayesian theory tells us that the best estimate of the hidden coin’s position would be to combine *prior* information about the coin’s position with the *sensory* information, or likelihood, about its current location. Furthermore, Bayesian theory tells us that they should be combined according to the relative reliabilities associated with each piece of information:

1 $${\text{X}}_{{{\text{est}}}} = {\sigma _{{\text{L}}}^{2}} /({\sigma _{{\text{L}}}^{2}} + {\sigma _{{\text{P}}}^{2}} )\upmu {\text{P}} + {\sigma _{{\text{P}}}^{2}} /({\sigma _{{\text{L}}}^{2}} + {\sigma _{{\text{P}}}^{2}} ){\upmu _{{\text{L}}}}$$

In our experiment, X_est_ is the participant’s estimated position of the coin, σ^2^_L_ and μ_L_ are the variance and mean of the likelihood/sensory information (here the spread and mean of the cloud of dots), and σ^2^_P_ and μ_P_ are the variance and mean of the prior (here the distribution of coin positions). For all Bayesian models fitted to the data (Models 4–7 in Table 1), Eq. (1) was used. (see “Methods” and SI for details). Note that σ^2^_P_/(σ^2^_L_ + σ^2^_P_) is the Bayesian weight on current sensory information, and if participants are behaving in a Bayes-optimal way then it should be identical to the obtained sensory weights.

**Table 1:** Model performance.

Models	# Free params	BIC	AIC
1. Senses only	0	− 97,099	− 97,107.7
2. Prior mean only	0	− 75,944	− 75,952.4
3. Previous coin location	0	− 61,522.3	− 61,530.7
4. Bayes opt. task parameters	0	− 98,493	− 98,501.6
5. Bayes opt. prior learned	0	− 98,256	− 98,264.5
6. Bayes opt. 2 priors total	2	− 101,395	− 101,421
7. Bayes opt. 2 priors per participant	120	− 103,652	− 104,680
8. Bayes opt. stress on slope	3	− 101,478	− 101,512
9. Bayes opt. stress on prior var	3	− 101,387	− 101,421
10. Bayes opt. stress on likelihood var	3	− 101,524	− 101,557

Represented, for each model, are the number of free (fitted) parameters, the Bayesian Information Criteria (BIC) and the Akaike Information criteria (AIC). The results were obtained using the full dataset. Lower values of BIC and AIC (here, more negative) suggest a better model fit. Free Params = Free parameters.

We also tested simpler models that do not combine prior and current information to see if they would better model participants’ data. Model 1 (the senses-only model) assumes that participants do not rely on prior information at all, and so their estimate at each trial of the coin location is just the centroid of the cloud of dots in that trial. Model 2 (the prior mean-only model) assumes that participants do not rely on current sensory information, and that their coin location estimates are always the mean of the prior, i.e. 0.5. Model 3 (previous coin location) is a heuristic model that assumes that a participant’s current estimate of the coin’s position is the coin position at the last trial (a different type of prior information). Note that these models are not considered here as Bayesian Optimal models, as they do not take both pieces of information into account. They could, in principle, also be framed as Bayesian models in which either the uncertainty associated with the prior information is much larger than the one associated with the sensory information (Model 1), or vice-versa (Model 2 and 3), but for simplicity we are just calling “Bayesian Models” models that use both prior and likelihood information.

In terms of Bayesian models, Model 4 (Bayes-optimal with task parameters) provides the Bayesian Optimal estimate using Eq. (1) and the experimentally-imposed prior variance values. However, it is very likely that participants’ subjective variances are not the experimentally imposed ones. This may be particularly relevant for the prior variance estimates, which are not known beforehand and not directly observable, and have to be learned. In Model 5 (Bayes-optimal with prior learned per trial) we account for learning and have the prior variance being updated trial by trial based on where the coin was. Model 6 (Bayes-optimal with 2 fitted Prior Variances Total) fits the two prior variances to the data instead of using the experimentally imposed prior variances, but assumes that the two prior variances are the same across all participants. Model 7 (Bayes-optimal with 2 fitted Prior Variances per Participant) estimates two prior variances per participant, as it assumes that there is individual variation in the perceived prior variances. All these models combine prior and likelihood information using the optimum prescribed by Bayesian statistics (Eq. (1)). See “Methods” and SI for additional model details and model simulation results (Supp. Figs. 1, 2).

**Figure 4:**
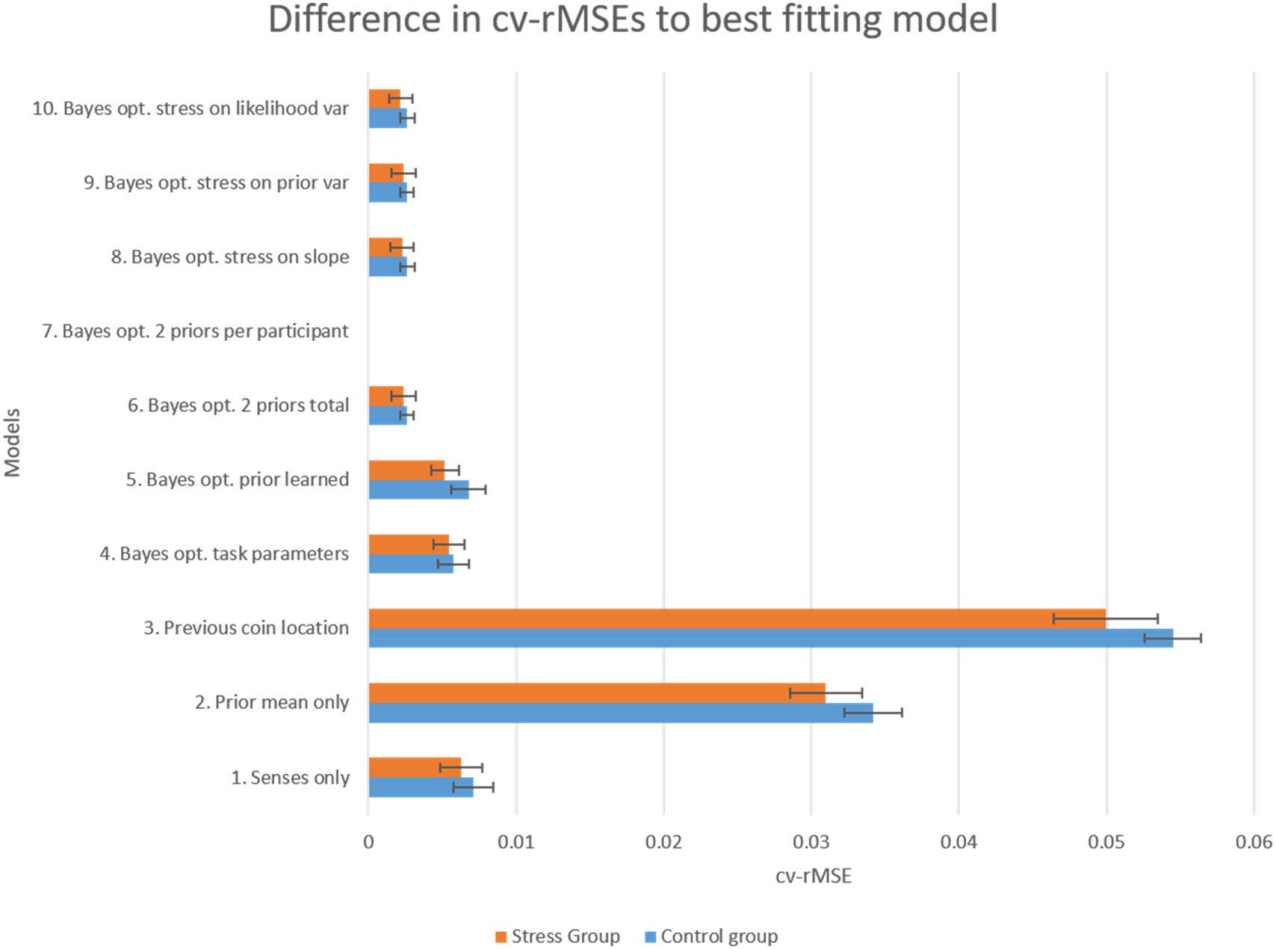
Model performance per group. Represented are the mean differences in the obtained cross-validated root mean squared errors (cv-RMSEs) between each model and the best performing model (here model 7), separated by group (see SI for details). Error bars represent the standard error of the mean.

We can see that, for the models fitted to the data and presented in Table 1, the Bayesian-optimal model that estimates two prior variances per participant gave the best fit (Model 7 in Table 1; Fig. 4). This model had the lowest BIC (more negative). In addition, the cross-validated root mean squared errors (cv-rMSEs) associated with this model are significantly lower than the ones obtained for the other models (p < 10^–8^ for all, Wilcoxon signed Rank test). This model was a good fit not only at the group level but even at the individual level (see SI and Supp. Fig. 1). The prior mean-only model (Model 2) and the prior coin position model (Model 3), on the other hand, had the worst model fits (significantly higher cv-rMSE, p < 10^–9^ for all, signed Rank test), with the previous coin position model performing even worse than the prior-mean only model (T(60) = 47, p < 10^–5^ Wilcoxon signed-rank p < 10^–9^, although one participant did seem to follow this strategy, see SI and Supp. Fig. 2 for details). The senses-only model (Model 1), although significantly better than the prior-only models (Models 2–3), was still worse than the Bayesian optimal models, suggesting once more that, although participants rely on sensory information, they do so in a way close to the Bayesian optimum, relying more on it when the sensory uncertainty is lower or their prior uncertainty is higher.

To understand if the best model fit depended on whether participants were in the not-stress or stressed group, we also analyzed separately the cv-rMSEs associated with each group and model. Looking at the results separated per group we find that the pattern of model fits was similar between groups, with Model 7 (Bayesian_2 priors per participant) giving the best fit, in either group, compared with the other models (lowest cv-rMSE, *p* < 0.01 for all, signed Rank test; see Table 1), and the prior-only models giving the worst fits. There was no difference in model performance between the two groups for any of the models tested (*p* > 0.1, rank sum test). Thus, the best model fit did not depend if participants were in the stressed or not-stressed group, and for both groups a Bayesian optimal model (Model 7) provided the best fit.

The fact that the model that gave the best fits estimated two prior variances per participant, one for the low prior uncertainty trials and one for the large prior uncertainty trials, allows us to analyze if indeed participants learned that these were two different prior uncertainties (see “Methods” for details). Overall, the estimated variances for the high prior uncertainty blocks were significantly higher than the estimated variances for the low prior uncertainty blocks, indicating that participants learned there were two different prior uncertainties and reacted accordingly (T(60) = 1484, *p* < 10^–4^, Wilcoxon signed-rank test). However, they were significantly higher than the experimentally imposed prior variances, suggesting that participants tended to overestimate the real prior variances and/or underestimate the sensory variance (T(60) > 1616, *p* < 10^–6^).

We can check if the obtained prior variances from Model 7 differed significantly per experimental group, as it would suggest different reliance on prior vs. sensory information. As outlined before, if acute stress increases reliance on sensory information, in this model this would show as an increased prior variance in the stressed group (given that a higher prior variance in the Bayesian model would lead to higher reliance on sensory information). If we look at the estimated prior variances per group, the same general pattern is observed, with the estimated variances for the large prior uncertainty trials being significantly higher than the ones for the smaller prior uncertainty trials (T(31) = 418, *p* < 0.001 (not-stressed); T(29) = 335, *p* = 0.011 (stressed), and also both variances higher than the experimentally imposed prior variances (T > 372, *p* < 0.001 for all, n = 31 and 29, Wilcoxon signed-rank test). There neither was a significant difference in the estimated prior variances between groups (d = 0.09, *W*(31,29) = 957, *p* = 0.8707), nor in the degree by which the high uncertainty prior variances differed from the smaller uncertainty ones (d = 0.4, *W*(31,29) = 984, *p* = 0.5740, Wilcoxon rank sum test). Together, this indicates that both groups were able to learn that there were two different prior variances, although these were inflated compared to the actual experimentally-imposed ones. In addition, the fact that we found similar estimated prior variances per group suggests once more a similar reliance on sensory information.

### Modelling stress

In addition to the models reported above, we fitted models that explicitly tested for the effect of stress. Model 8 (Bayes-optimal, stressed on slope) is similar to Model 6 (Bayes-optimal with 2 fitted Prior Variances Total) but includes an additional dummy variable that models the effect of stress on the sensory weight. Model 9 (Bayes-optimal, stressed on prior variance) has the effect of stress directly affecting the estimated prior variance instead and Model 10 (Bayes-optimal, stressed on likelihood variance) has stress affecting the likelihood variance (see SI for details). Furthermore, besides fitting these models on a participant-by-participant basis, we also used a multi-level modelling approach, with stress as a fixed effect and participants as a random effect (see SI for details). For all models tested, the addition of the stress dummy variable did not significantly improve the average cross-validated rMSEs in comparison to an identical model without it (no significant improvement over Model 7, t(59) < 0.92, *p* > 0.37 for all, see Table 1 and Fig. 4). These results match the results presented above, suggesting that our stress procedure did not significantly affect participants’ sensory weights or their prior and likelihood variance estimation.

We can also use multi-level (hierarchical) modeling to directly test if the experimental procedure affected participants’ sensitivity to changes in prior and likelihood uncertainty. If we use the experimental manipulation (stressed) as a fixed effect predictor variable of participants’ sensitivity to changes in prior or likelihood uncertainty, and participant as a random effect, we find that, for both cases, there is a significant intercept value but no significant effect of the experimental manipulation. This indicates once more that stress did not affect how participants’ reacted to changes in prior and likelihood uncertainty.

## Discussion

In this study, we addressed the question of whether and how stress affects low-level decision-making and, specifically, if stress would affect the reliance on sensory vs. prior information. To this end, participants had to make a guess about a target’s position, and could incorporate both prior information (i.e., where a previous target was located) and current sensory information (i.e., the ‘noisy’ sensory information about the target’s position) when making that decision. In Bayesian framework, information from bottom-up, i.e., the sensory input is referred to as *likelihood*, whereas previous knowledge that in integrated with such data is referred to as *prior*^14^. The mathematical models allow computing whether participants put more weight on the one or the other. We randomly allocated participants into two groups, one that would be exposed to a stressful manipulation before doing the task (the socially evaluated cold pressure task; SECPT^18^) and other that was exposed to a non-stressful manipulation. Based on a currently discussed proposal, we expected stress to induce a shift to bottom-up processing^7^. In the context of our task, we hypothesized that this shift would result in an increase in reliance on sensory information. Contrary to our expectations, we did not find an effect of stress on the average reliance on sensory information. Both participant groups were equally sensitive to changes in prior or likelihood uncertainty, and reacted to these changes in a way qualitatively and quantitatively predicted by Bayesian statistics.

One may argue that the lack of differences may be due to ceiling effects, as many participants showed average sensory weights above 0.9. To check on this, we analysed the sensory weights associated with the small prior uncertainty large likelihood uncertainty condition (pL), given that this condition had the lowest average sensory weights. There were no significant differences in the sensory weights between the stressed and not-stressed conditions (see Supplementary section). This suggests that although ceiling effects may have played a role, they are likely not the only explanation for the lack of effects observed.

Previous studies have shown that under stress, both the detection of schema-congruent information and the neural ensemble underlying schema-related processing are affected^21^. A schema is a complex mental structure that allows interpreting incoming sensory data based on prior knowledge about events, shapes and statistics of actions, objects and scenes^22^. Thus, such impairments suggest that prior knowledge is more or even completely eschewed under demanding, stressful conditions. This aligns with a plethora of studies reporting impaired memory retrieval under stress^11^. A shift from top-down to bottom-up information, i.e., away from internal processing and expectations to actual events happening in the environment seems adaptive in stressful conditions in many ways: First, the stress reaction may suggest that something about the way we interacted with the environment was faulty, thus reliance on prior models may be even harmful. Second, in order to quickly react to a possibly dangerous situation, the environment needs to be scanned and analyzed carefully. Weak signals may turn out lethal threats, e.g., detecting the tail of a tiger behind a tree, and their discovery possibly lifesaving. Indeed, there is preliminary evidence that stress promotes the detection of bottom-up information in the environment^7^.

How can the present data be reconciled with the hypothesis of resource re-allocation under stress, i.e., an enhanced focus on current information from bottom-up? First of all, it is important to mention that stress can be roughly divided into two time scales that differ with regard to neural and physiological consequences. The acute stress phase is dominated by the release of catecholamines (adrenaline and noradrenaline), and is usually linked to the duration of the actual stressor^23^. This rapid stress reaction is responsible for the regulation of the cardiovascular and respiratory system, and is characterized by activation of the sympathetic nervous system, preparing the organism for a *flight-or-fight* mode^24^. During the later phase, after approximately twenty to thirty minutes, corticotropin-releasing-hormone is released from the hypothalamus, which ultimately leads to the synthesis and release of glucocorticoids^25^. The role of glucocorticoids is to aid the organism to return to homeostasis, e.g., by reinstating energy resources^26^. In the current study, the experiment started twenty minutes after the stress induction, i.e., when cortisol, an important glucocorticoid, is supposed to peak^27^. It is possible that an enhanced processing of bottom-up information is linked to the acute, adrenergic phase only, i.e., the time window when analysis of the environment is required to happen quickly enough to be helpful. The focus on the extended rather than acute stress reaction is certainly a limitation of the present study. However, the deteriorating effects of stress on using schema-related information had been demonstrated regardless of the timing of the stressor^28^. It may be that whether or not prior information is used depends on various factors, and identifying those factors may be an important avenue for future research. It may also be interesting to probe for neural or neurophysiological correlates of priors and their modifications during stressful episodes. For instance, it has been demonstrated that if specific faces are more likely to occur, i.e., have a higher prior probability; the brain activity in the respective areas is higher before their actual physical occurrence, as opposed to faces with a lower prior probabililty^29^. If prior information tends to be eschewed under stress, such differences could be less pronounced, and instead brain activity would be higher during processing of the actual stimulus. Another explanation could be that *only* salient sensory information (in contrast to all information) is processed more strongly under stress, and therefore leads to the hypothesized shift to sensory or bottom-up information. As none of our stimuli were more or less salient than others, and were also affectively neutral, this account cannot be addressed in the context of our study design. Future work is required to examine the role of the stimulus characteristics that modify or do not modify low-level decision making under stress.

Our study replicates previous findings: participants, in a sensor decision-making task, behave in a way consistent with the optimum prescribed by Bayesian statistics^15–17,19^. The model that best fit the data was a Bayesian optimal model. However, it is worth noting that the best fitting model was one in which the prior variances could be estimated per participant, and the estimated prior variances were significantly higher than the experimentally imposed ones. This is in agreement with studies suggesting that participants may tend to overestimate the prior uncertainties, potentially because it may take them longer to estimate the real prior variances^30^ or they may not ever fully learn it if the task is too complex^31^. Future studies should look further to this issue, to better understand people’s limits in learning the parameters associated with Bayesian decision-making.

## Methods

### Participants

Sixty participants (mean age 24.7 years, SD 2.7; 32 males) were recruited by flyers displayed at the University of Hamburg. The number was based on prior studies using the Bayesian Decision Making task, showing robust effects of likelihood and prior manipulation at comparable sample sizes^17^. The participants were selected based on a standardized telephone interview, which served to exclude past or current mental or neurological disease and a BMI < 18 kg/m^2^ or > 26 kg/m^2^. Further exclusion criteria were any sort of medication, smoking, alcohol or drug abuse as well as intake of hormonal contraceptives in women (during women menses, female participants were not tested). Informed written consent was provided by all participants before taking part in the experiment. The study protocol was approved by the medical ethics committee Hamburg and in accordance with the Declaration of Helsinki. Participants were paid 60 Euro for their participation and were randomly assigned to one of two experimental groups, stress versus control.

### Stimuli and task

In order to assess how participants make (perceptual) decisions under stress, we adapted a version of a visual decision-making task^17^. On the computer screen, blue dots were displayed on a grey background, with additional black dots serving as background stimulation (see Fig. 2). The blue dot-cloud was drawn from a Gaussian distribution centered at the true target position. Participants’ task was to guess the position of a hidden target (they were told the dots are splashes from a coin thrown into a lake, and the imagery person who “threw” the coin aimed hitting the center, and they had to guess the position of this coin within the blue dot-cloud, and they could use both the likelihood, obtained from the displayed dots distribution, and the prior, obtained from the distribution of previous target coin positions. Participants would signal their estimated coin position by placing a “net” (vertical rectangle bar) where they thought the coin was and pressing a button. As the net covered the full screen in the y-direction, participants only had to estimate the x-position of the coin. Once they pressed the button, the true hidden position of the coin in that trial was revealed (during 1.5 s). If the final net position was covering at least half of the hidden coin, participants would get a point added to their total score. The initial net location varied randomly from trial to trial. Parameter values were similar to the ones used in^17^: The distribution from where the coin was drawn (the prior distribution) was a Gaussian distribution that had the mean at the center of the screen (0.5 in screen coordinates) and the variance was either small (σ^2^_p_ = 0.025^2^) or large (σ^2^_P_ = 0.085^2^ in unit-less screen coordinates). The mean of the prior distribution was given to the participants (“the thrower is aiming at the center of the screen”). The cloud of blue dots (the likelihood distribution) was composed of 5 blue dots, and each dot was taken from a Gaussian distribution where the mean was the real position of the coin on that trial and the variance was either small (σ^2^_l_ = 0.06^2^) or large (σ^2^_l_ = 0.15^2^). Hence, while the prior uncertainty of the coin position had to be learned, the spread of the blue dots was directly observable at each trial. The experiment was divided in 4 blocks of 150 trials each, for a total of 600 trials in the experiment. Within each block the prior uncertainty was constant, but it changed between blocks (2 types of prior variance * 2 block repetitions). Contrary to our previous study^17^, the 4 prior blocks could occur in any combination (previously, the prior blocks were always alternating, so that there were never 2 blocks of the same prior uncertainty in a row). Participants were given feedback at the end of each trial; no additional reward was provided for correct answers (besides increasing their score on the game).

The task was programmed and presented using the Psychophysics toolbox^32^ for MATLAB (The Mathworks), took place in a dimly illuminated room, and lasted for approximately 30 min. Participants received a training of 6 trials before the experiment started, which could be repeated if necessary.

In addition, subjective chronic stress, depressive mood, general and social anxiety were assessed by letting participants complete the Trier Inventory for the Assessment of Chronic Stress, TICS^33^, the Beck Depression Inventory, BDI^34^, the State–Trait Anxiety Inventory, STAI^35^ and the Liebowitz Social Anxiety Scale^36^. All of these control measurements were completed before the experiment. TICS, BDI and the questionnaire about the subjective experience of the stress manipulation were assessed during the 20 min waiting time after the SECPT manipulation, and before the decision-making experiment. The entire experiment, including questionnaires, stress manipulation, and task, lasted about 1.5 h.

### Stress induction

To induce stress, we used the socially evaluated cold pressure task (SECPT)^18^. In the stressed group, participants had to immerse their hand into ice water (0–2 °C) and held it there for three minutes. During this time, participants had to look into a camera and were additionally observed by an unfriendly, non-supportive experimenter in a white coat. The experimenter made notes during his/her observation. For the control group, the water was warm (35–37 °C), there was no camera, and the experimenter was friendly and casually dressed. All testing took place in the afternoon (13:00–18:45) to control for the diurnal rhythm of cortisol. The decision-making experiment started approx. 20 min after the SECPT manipulation.

### Cortisol samples

We obtained saliva samples using Salivette collection devices to measure the stress hormone cortisol (Sarstedt, Germany). First, all samples were stored at − 18 °C (− 0.4 °F) immediately after the experiment. When the study was completed, all samples were thawed for biochemical analysis, and the fraction of free cortisol was assessed using a commercially available chemiluminescence immunoassay (IBL, Tecan Trading AG, Switzerland). The cortisol samples were obtained (i) immediately after the participant entered the room, i.e., before the questionnaires and the decision-making experiment, (ii) 20 min after the stress manipulation with SECPT, and (iii) at the very end, i.e., when participants completed all questionnaires and the entire decision-making experiment.

### Computational modeling of behavior

See supplementary information.

### Statistical analysis

See supplementary information.

## Supplementary information

Supplementary Information.

## Data Availability

The datasets and the code generated during and/or analyzed during the current study are available from the corresponding author on reasonable request.
