# Supplementary material for: Bayesian decision-making under stress-preserved weighting of prior and likelihood information
Source: Sci Rep. 2020 Dec 8;10:21456. doi: 10.1038/s41598-020-76493-5 (PMC7722735; doi:10.1038/s41598-020-76493-5)
Supplement: Supplementary file 1 — Supplementary Information. [file 41598_2020_76493_MOESM1_ESM.docx]

**Supplementary information**

**Computational modelling of behavior**

***Bayesian modelling of behavior***

Bayes rule provides us with an optimal way to combine prior and current sensory information (or likelihood), in a way that reduces overall uncertainty^15,16,17,18^:

X_posterior estimate_=σ^2^_L_/(σ^2^_L_+ σ^2^_P_)μ_P_+σ^2^_P_/(σ^2^_L_+ σ^2^_P_)μ_L_ (1)

In our experiment, X_posterior estimate_ is the participant’s estimated position of the coin, σ^2^_L_ and μ_L_ are the variance and mean of the likelihood/sensory information (obtained from the displayed cloud of dots), and σ^2^_P_ and μ_P_ are the variance and mean of the prior (obtained from the distribution of coin positions). The mean of the prior (μ_P_), in our task, was the mean of the distribution from which the coin was drawn, specifically, 0.5 in screen coordinates, i.e. the center of the screen). The mean of the likelihood (μ_L_) here is the centroid of the displayed cloud of dots, and as such it can change from trial to trial.

If we perform a simple linear regression on the participant’s estimated coin position at each trial as a function of the centroid of the displayed cloud of dots in that trial, the slope of this regression gives us the *sensory weight*, a measure of by how much the participant is relying on likelihood/sensory information. If participants perform according to the Bayesian Optimum portrayed in *equation (1)*, then this slope, or sensory weight, should be equal to the perceived σ^2^_P_/(σ^2^_L_+ σ^2^_P_). Slopes close to 1 indicate that participants are relying almost exclusively on the sensory information (for example because their perceived prior variance is much higher than their perceived likelihood variance), while if they are close to 0 they indicate that participants are not relying on current information (hence potentially relying more on prior information, although this is not necessarily the case). This slope, thus, shows the relative reliance on sensory information, i.e. the *sensory weight*. If we assume that participants are using only sensory information or prior information (e.g. excluding random behavior), then the weight on prior information is just *1 - sensory weight* ^15,17,18^. Note that, by construction, the sensory weight σ^2^_P_/(σ^2^_L_+ σ^2^_P_) has to be between 0 and 1. However, the actual obtained slopes may not be between 0 and 1, and if they are very far from this range it suggests that participants are far from what would be expected by Bayesian statistics.

There were two participants with negative slopes, both from the stress group (participant #8 and #26, see supplementary results). The data from these participants also satisfied the criteria of hard outliers (according to Tukey’s interquartile range criteria). Performing the results shown in this paper without these two participants gives rise to qualitatively similar results, albeit with general lower mean squared errors and lower variability.

***Fitted Models***

A brief summary of each model is described in the results section. Models were fitted using MATLAB. Models in which free parameters had to be fit (models 6-10) used the Matlab function *fmincon*. Models 1, 4-10 use as variance of the likelihood σ^2^_L_= variance (cloud of dots)/ number of dots, thus for our experiment σ^2^_l_=0.06^2^/5 for the small likelihood uncertainty trials and σ^2^_L_ = 0.15^2^/5 for the large likelihood uncertainty trials. Performing the models using the actual standard deviation of the cloud of dots at each trial gives similar results (same relative performance between the models). Note that the participant’s actual subjective likelihood uncertainty could have been different, but we choose the experimentally imposed ones for simplicity. For models 2 and 4-10 the mean of the prior (μ_P_) used was assumed to be the real (experimentally-imposed) one, namely 0.5 (the center of the screen), given that participants were explicitly told that the imagery person who “threw” the coin aimed hitting the center. The mean of the likelihood was the X-centroid of the displayed cloud of dots at each trial (obtained by averaging the x-positions of the 5 likelihood dots). The variance of the prior (σ^2^_P_) was calculated differently for different models (see below).

*Models without free parameters:*

Model 1 (senses-only model) assumes that the weight on sensory information is 1, hence each participant’s estimate of the coin location is equal to the centroid of the cloud of dots in that trial.

Model 2 (prior mean-only model) assumes that the weight on sensory information is 0 (and the weight on prior information is 1), thus participants’ coin location estimates are always the experimental mean of the prior (μ_P_), i.e. 0.5.

Model 3 (previous coin location) uses the mean of the prior (0.5) as the estimated coin location for the first trial, and after that uses whatever the coin position was the trial before. It also only uses prior information but a different type of prior information.

Neither of these models would be considered Bayesian models, as they do not take both pieces of information into account. However, if we think about Bayesian models as a way to frame any model and see how participants differ from this model, we can consider these models as Bayesian models but in which either the prior variance (for model 1) or the likelihood variance (for model 2 and 3) are much larger than the other variance, making the sensory weight (σ^2^_P_/(σ^2^_L_+ σ^2^_P_)) essentially 1 (for model 1) or 0 (for models 2 and 3). However, for simplicity we are just naming “Bayesian Models” models that combine both prior and likelihood information.

Model 4 (Bayes-optimal with task parameters): to estimate where the participant would have placed the net at each trial (their supposed best estimate of the coin’s position in that trial) it uses equation (1) and the experimentally-imposed prior variance values, namely σ^2^_p_=0.025^2^ for the small prior uncertainty trials/blocks andσ^2^_P_ = 0.085^2^ for the large prior uncertainty trials.

Model 5 (Bayes-optimal with prior learned per trial): Similar to model 4 but the prior variance is learned trial to trial. Specifically, the variance at each trial is calculated by:

$${\sum_{1}^{t-1} ({{coinPosition}_{t-1}-\mu_{p})}^{2}}/{(t}-1)$$

Where μ_P_ is the experimental mean of the prior (0.5) *t* is the current trial number, and *coinPosition_t-1_* is the coin position at the last trial. For the first trial, given that participants haven’t seen any coin location yet, the prior variance is set as 0.5^2^.

*Models with free parameters:*

Model 6 (Bayes-optimal with 2 fitted Prior Variances Total) also uses equation (1) but instead of using the experimentally imposed prior variances (like in Model 4), it fits the two prior variances to the data. It assumes that the two prior variances (one for the small prior uncertainty trials and one for the large prior uncertainty trials) are the same across all participants, regardless of the group. The estimated variances were constrained to be between 0 and 1, given that a variance has to be a non-negative number, and given that the total screen size is 1 (hence 1 would be the logical maximum prior variance). However, constraining the variances to be between 0 and *+Inf* gives the same results*.*

Model 7 (Bayes-optimal with 2 fitted Prior Variances per Participant) is identical to Model 4 but estimates two prior variances per participant, as it assumes that there is individual variation in the perceived prior variances. Hence, there are a total of 120 free parameters estimated for this model (2 prior variances per participant * 60 participants). Estimated variances were constrained to be between 0 and 1.

Model 8 (Bayes-optimal, stress on slope): It is identical to Model 6 but it includes an extra free parameter that is added to the slope/sensory weight (σ^2^_P_/(σ^2^_L_+ σ^2^_P_)) and corresponds to the putative effect of the stress procedure on the sensory weight. This free parameter is multiplied by a dummy variable that is 0 for a participant that belongs to the control group and 1 for a participant that belongs to the stressed group.

Model 9 (Bayes-optimal, stress on prior variance): Is identical to Model 8 but the free parameter (*dummy) is added directly to the estimated prior variance instead.

Model 10 (Bayes-optimal, stress on likelihood variance): Is identical to Model 8 but the free parameter (*dummy) is added directly to the likelihood/sensory variance instead.

Models 8,9, and 10 were, in addition, also fitted using a hierarchical modelling approach, using the nlmefit function in matlab (see below additional details).

***Model comparison***

To compare model performance overall, we used the Bayesian Information Criterion (BIC) and Akaike Information Criterion (AIC). The BIC and AIC take into account both the log-likelihood associated with each model but also introduce a penalty for adding model parameters. This is done in order to minimize overfitting. For each model, the number of free parameters is shown in Table 1. Note that the number of observations is equal to the number of trials*number of participants, hence it is 36000 (600*60), thus much larger than the number of parameters used in any of the models (even in Model 7). A model with a lower BIC/AIC is generally preferred.

Besides comparing BIC/AIC, we also compared the root mean squared errors (rMSEs) obtained by each model for each participant. In this way, we are able to look at how the models may perform across groups. However, because the models have different numbers of free parameters, using just the rMSE directly would not be appropriate (as lower rMSEs could just be due to overfitting). Instead, we used the cross-validated rMSE. For this, we fitted the model using only the odd trial numbers, and tested it on the even trial numbers. Thus, the rMSEs reported are the rMSEs obtained from the even trials (cross-validated rMSEs). This gave us a cross-validated root mean squared error (cv-rMSE) associated with each model and participant. In this way, using the cross-validated rMSEs controls for model complexity.

For both model 3 and 5, when cross-validating the values are still calculated based on all the trials (even and odd), but the mean squared errors were only calculated using the even trials. This was done so that cross-validated mean squared errors (cvMSE) are not artificially high. For the models with free parameters, only the odd trials are used to fit the parameters, and the cvMSE values are based exclusively on the even trials.

***Statistical analysis***

Data was analyzed using *MATLAB*.

Comparison between groups (e.g. participants in the stressed vs. not-stressed group) was made with a 2-sample t-test (t), or, if assumptions for the t-test weren’t met, the Wilcoxon Rank-Sum test (W). Comparison within groups (e.g. different model performances within the same group; or comparing with a mean/median of 0), was made with a paired t-test or a Wilcoxon Signed-Rank test (T). All reported tests are 2-tailed.

General effects of prior, likelihood (sensory evidence) and group on the sensory weights were analyzed with a 3-factors repeated-measures ANOVA, with prior, likelihood and group as fixed factors and all the 2-way interactions modelled.

Sensory weights, sensitivity to prior and likelihood uncertainty were modeled similarly to a previous study^10^. Namely:

*General sensory weight*: was calculated as the slope of the linear regression of participant’s final net position (i.e. their estimated coin position), as a function of the centroid of the cloud of dots (in the x-direction).

*Sensory weight per condition*: For each condition (small prior and likelihood uncertainty (pl); small prior uncertainty, large likelihood uncertainty (pL); large prior uncertainty, small likelihood uncertainty (Pl); and large prior and likelihood uncertainty (PL)), we obtained the associated trials (75 trials per condition), and did a linear regression of the participant’s final net position (i.e. their estimated coin position), as a function of the centroid of the cloud of dots. The slope of this regression was the sensory weight associated with that condition, per participant (*sw*).

*Sensitivity to prior uncertainty*: we calculated it as the difference between the sensory weights of the large prior uncertainty conditions and the sensory weights in the small prior uncertainty conditions (i.e. ((swPl +swPL )-(swpl+ swpL))/2).

*Sensitivity to likelihood uncertainty*: we calculated it as the difference between the sensory weights of the large likelihood uncertainty conditions and the sensory weights in the small likelihood uncertainty conditions (i.e. ((swpL +swPL )-(swpl+ swPl))/2).

*Performance*: we calculated the proportion of correct trials, per condition, as the number of times the participant was able to guess the position of the coin, divided by the total number of trials in that condition.

**Multilevel (hierarchical) modelling**. Multilevel (hierarchical) modelling was done in Matlab using the function *fitlme* as well as the function *nlmefit*. Stress was modelled as a fixed effect and participant number as a random effect (both for the intercept and the slope). The stress parameter was multiplied by a dummy variable that was 0 if the trial was from a participant in the not-stressed group and 1 if the participant was in the stressed condition. When calculating the effect of stress on the sensitivity to prior or likelihood sensitivity, we added stress as a fixed effect and participant number as a random (intercept) value.

For the multi-level approach of Model 8,9, and 10, which include stress as a fixed effect and participant number as a random effect (both for the intercept and the slope), the cross-validated model prediction results were obtained by using participant-level parameters that are a sum of both the population-level parameters and the individual-level effects. The parameters were fitted using only the odd trials, and the cv-rMSE values were calculated based only on the even trials.

**Effect sizes and Power analysis**. Power analysis were performed using the software GPower 3.1^36^. Effect size was calculated using the Cohen’s d effect size, with formula:

$$\frac{\bar{X}_{2}-\bar{X}_{1}}{s_{pooled}}$$

Where $\bar{X}_{2}$ represents the sample mean associated with group 2 (here the stressed group), $\bar{X}_{1}$ is the mean associated with group 1 (here the control group), and s_pooled_ is the pooled standard deviation.

**Supplementary Results**

***Influence of previous coin position***

To analyze if participants were instead following a strategy of just placing the net wherever the coin had been the trial before, we did a linear regression predicting a participant’s net position per trial (their estimate of the hidden coin position) as a function of both the centroid of the cloud of dots in that trial and the coin position the trial before. We found that both the centroid of the cloud of dots (T_60_ = 1828, p<10^-10^, Wilcoxon signed-rank) as well as where the coin was the trial before (T_60_ = 1590, p<10^-6^) were significant predictors of the participant’s estimated position. However, on average the centroid of the cloud of dots was a much stronger predictor compared to the previous coin position (T_60_ = 1778, p<10^-9^). Indeed, for all but 2 participants the centroid of the cloud of dots exerted a very significant and stronger influence compared to the previous coin position (see paragraph below). There was also no significant difference between the experimental groups on the effect of the prior coin position (*W*(31,29) = 962, p = 0.813, Wilcoxon rank-sum test). Thus, overall, while the prior coin location may have exerted some influence, participants’ behavior cannot be explained by simply having followed a strategy of placing the net wherever the coin was the trial before.

***Model fits – per participant***

While we have been looking at average effects, there are individual variations. For 58 out of the 60 participants, the centroid of the cloud of dots exerted a significant and stronger influence compared to where the coin was the trial before. In line with this, **Model 7** (Bayes Optimal with 2 prior variances per participant) did the best job in predicting participants’ estimated coin position (see Supp. Fig 1). One exception was participant (#26), where the prior coin location exerted a stronger influence (which can be seen by the better fit of Model 3, Supp. Fig 2). The only other exception was participant #8. For this participant neither the prior coin position nor the centroid of the cloud of dots exerted a significant effect. Also, looking at this participant’s distribution of net positions we see that while for the other participants these are concentrated around the mean of the prior (the center of the screen, 0.5), for this participant the estimated coin positions were spread more or less evenly across the screen, suggesting that this participant played randomly (see Supp. Fig 3). Doing the analysis in this paper without these participants leads to similar results. Thus, although some individual variation in model fit does exist, for the vast majority of participants a Bayesian optimal model gave the best fit, even at the individual level.

***Ceiling effects***

As several participants have average sensory weights above 0.9, these participants seem to have relied almost exclusively on the current sensory information. We analysed the sensory weights associated with the small prior uncertainty large likelihood uncertainty condition (pL), given that this condition had the lowest average sensory weights (sw_pL_ = 0.57+-0.03, mean+-se). There were no significant differences in the sensory weights between the stressed and not-stressed conditions (d = 0.03, *W*(31,29) = 921, p=0.7226, Wilcoxon rank-sum test).

**Supplementary Figures**


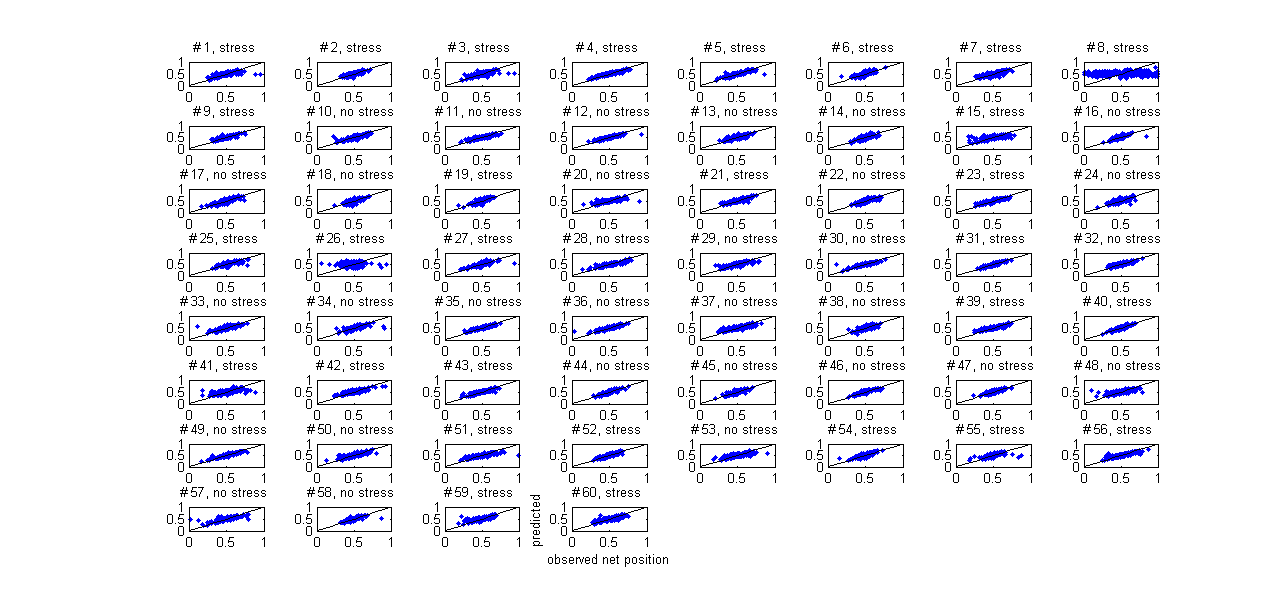


Supplementary Fig. 1. Individual model fits, for Model 7. Represented in the x-axis are the observed final net positions (which signal a participant’s estimation of the hidden coin location), and in the y-axis the net position as predicted using Model 7 (Bayes-optimal with 2 fitted Prior Variances per Participant), per participant. Each blue dot represents a trial. The diagonal black line represents the unit line. We can see that overall (except for participants #8 and #26), the points are close to the unit line, indicating a good model fit. Each subplot is a participant, and the title indicates if the participant was part of the stress or not-stressed (control) group. Note that, although the figure appears tilted, it is not in fact so.


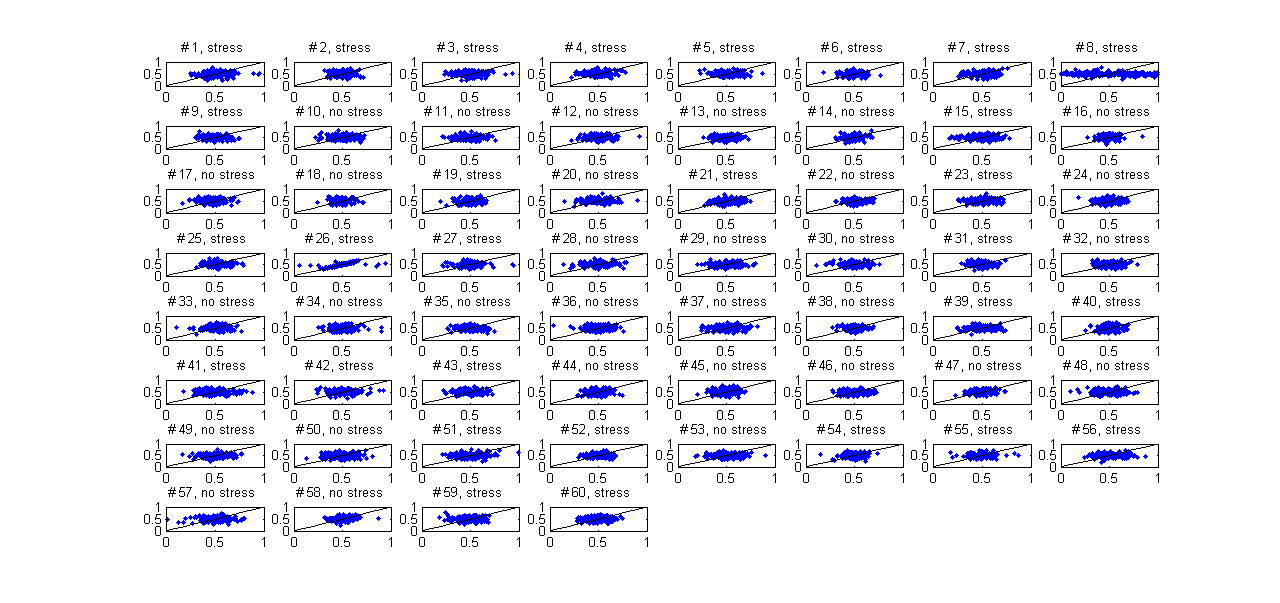


Supplementary Fig. 2. Individual model fits, for Model 3. Represented in the x-axis are the observed final net positions (which signal a participant’s estimation of the hidden coin location), and in the y-axis the net position as predicted using Model 3 (coin previous trial), per participant. Each blue dot represents a trial. The diagonal black line represents the unit line. Note that here, except for participant #26, for the majority of the participants the model gives a worse model fit (compared to model 7).


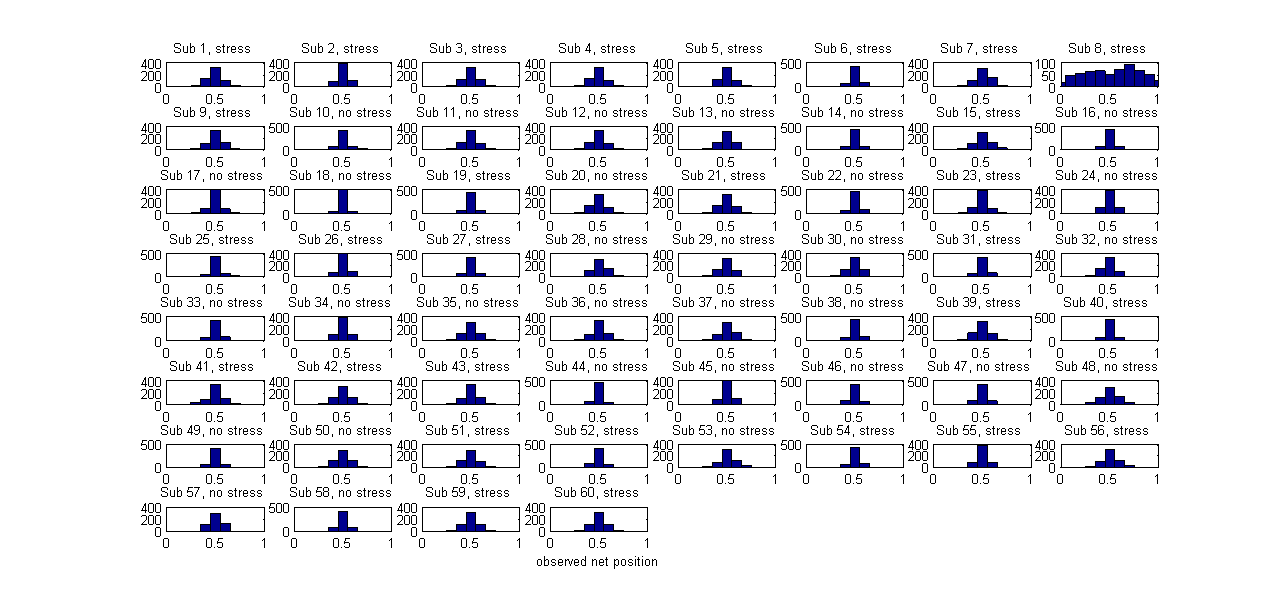


Supplementary Fig. 3. Histogram of observed net positions, per participant. We can see that for all participants except 1 (participant #8), the majority of participant’s final net positions (i.e. estimated coin positions), per trial, are centered around 0.5 (the center of the screen and the mean of the prior).
